# Datasets of seed mucilage traits for *Arabidopsis thaliana* natural accessions with atypical outer mucilage

**DOI:** 10.1038/s41597-021-00857-3

**Published:** 2021-03-09

**Authors:** Mireille Cambert, Adeline Berger, Christine Sallé, Stéphanie Esling, Delphine Charif, Tudel Cadoret, Marie-Christine Ralet, Helen M. North, Corinne Rondeau-Mouro

**Affiliations:** 1 INRAE, UR1466 OPAALE, 17 avenue de Cucillé, CS 64427, 35044 Rennes Cedex, France; 2 Institut Jean-Pierre Bourgin, INRAE, AgroParisTech, Route de Saint Cyr, RD10, 78000 Versailles, France; 3 INRAE, UR1268 BIA, 3, Impasse Yvette Cauchois, CS 71627, 44316 Cedex 3 Nantes, France

**Keywords:** Plant physiology, Polysaccharides

## Abstract

The seeds of *Arabidopsis thaliana* become encapsulated by a layer of mucilage when imbibed. This polysaccharide-rich hydrogel is constituted of two layers, an outer layer that can be easily extracted with water and an inner layer that must be examined *in situ* in order to study its properties and structure in a non-destructive manner or disintegrated through hydrolysis or physical means in order to analyze its constituents. Mucilage production is an adaptive trait and we have exploited 19 natural accessions previously found to have atypical and varied outer mucilage characteristics. A detailed study using biochemical, histological and Time-Domain NMR analyses has been used to generate three related datasets covering 33 traits measured in four biological replicates. This data will be a rich resource for genetic, biochemical, structural and functional analyses investigating mucilage constituent polysaccharides or their role as adaptive traits.

## Abstract (summary)

**Table:** 

Measurement(s)	intrinsic viscosity • galacturonic acid • seed and inner mucilage layer width • Transverse Spin Relaxation Time • Transverse Spin Relaxation intensity • mucilage • neutral sugar content • mass per seed • sample mass
Technology Type(s)	size-exclusion chromatography • colorimetry • immunolabeling method • confocal microscopy • nuclear magnetic resonance spectroscopy • Time-domain NMR • biochemical, histological and Time-Domain NMR analyses • biochemical method using orcinol • microbalance
Sample Characteristic - Organism	Arabidopsis thaliana

Machine-accessible metadata file describing the reported data: 10.6084/m9.figshare.13320593

## Background & Summary

Seeds of a number of plant species, including Arabidopsis (*Arabidopsis thaliana*), become surrounded by sticky mucilage when imbibed. A range of roles has been suggested for this polysaccharide-rich hydrogel, such as aiding germination, dispersion, seedling growth or interaction with soil microorganisms reviewed by Yang *et al*.^1^. In the reference accession for the model plant Arabidopsis, Columbia (Col-0), the major component of mucilage is the pectin rhamnogalacturonan I (RG-I), which is organized in two distinct layers that differ in their polysaccharide composition and structure^2^. This suggests that the outer water-soluble and inner adherent layer could perform different ecophysiological functions^2^.

Although Arabidopsis is used widely as a model for geneticists, it is a widespread weed whose native range covers most of Europe to central Asia. As mucilage is an adaptive trait its functional advantages are likely to influence the dynamics and evolution of natural Arabidopsis populations. Natural variation in the outer water-soluble layer of Arabidopsis mucilage was recently reported for 306 natural Arabidopsis accessions^3,4^. Large variations were observed in the amount and properties of the constituent polysaccharides. Nonetheless, the composition of the outer mucilage layer was stable between genotypes with RG-I always being the major constituent of outer mucilage. Analysis of the inner mucilage layer is more complex as the polysaccharides are tightly adhered to the seed surface and hydrolysis of the biopolymers into fragments is required. To date, the detailed composition of the inner mucilage layer has only been determined for a limited number of accessions used to generate induced mutant collections (Col-0, Col-2)^2,5^. As the inner mucilage layer can be observed using the cytochemical stain ruthenium red the visual aspect of inner mucilage was previously examined for 280 accessions^6^. This method identified fifty variants that differed in the size of the inner mucilage layer. Observation with ruthenium red can, however, only give an indication of major differences in the width of the inner mucilage layer and this is not necessarily an indication of more or less polysaccharides as the hydrophilic properties, molar mass and conformation of the pectin polymers can alter the volume they occupy^7,8^. Moreover, seed size can vary between natural variants and the volume of the mucilage layer may appear bigger or smaller due to these differences. Furthermore, loss of adhesion of inner layer pectin in the *muci70* mutant, was recently proposed to be linked to modified macromolecular characteristics as outer mucilage RG-I polymers were shorter in this mutant^4^, which suggests that polymer length contributes to adhesion through intermolecular entanglement. This highlights that much is still unknown concerning the physicochemical requirements for the formation of the inner layer.

To study the variability in mucilage traits in more detail, we have carried out a detailed characterization of both inner and outer mucilage traits for 19 natural variants identified previously as exhibiting atypical outer mucilage macromolecular properties^4^, these included the reference accession Col-0 (Table 1).

**Table 1:** Collection site and atypical outer mucilage traits for the Arabidopsis accessions used to generate dataset.

Versailles identification number (AV)	Accession name	Country of origin	Collection site co-ordinates (latitude/longitude)	Atypical Trait(s)
13	Hag-2	FRA	^*^49.675771/-1.800928	***GalA, NS*** and IV
77	Bla-2	ESP	^§^41.677605/2.792187	Rh
136	Kb-0	GER	^§^50.180479/8.516972	IV
166	Cvi-0	CPV	^§^15.064698/-23.7323	***GalA, IV*** and ***Rh***
167	Pn-0	FRA	^§^48.068054/-2.967396	Mp and Rh
178	Alc-0	ESP	^§^40.487771/-3.363247	Mp and Rh
**186**	**Col-0**	**POL**	^§^52.738911/15.237236	IV
254	Hiroshima	JPN	^§^34.513686/133.361435	***IV***
257	Sakata	JPN	^§^38.942641/139.831009	***GalA, NS*** and ***IV***
258	Tokushima	JPN	^§^34.085251/134.554253	***IV***
259	Yamagata	JPN	^§^38.476384/140.360412	***IV***
261	9481B	KAZ	^#^38.587464/68.787689	Rh
301	Cvi-2	CPV	^§^15.075306/-23.600464	***IV, Rh*** and ***Rg***
335	K-oz-3	RUS	^*^51.3333/82.1833	NS and Rh
397	Sq-8	UK	^*^51.25/0.41	***NS, GalA*** and Mp
456	Al-0	DEN	^§^55.19226/9.014096	Mp
472	Fl-3	FIN	^#^60.184755/24.937363	GalA, Mp, Rh and Rg
517	Ts-5	ESP	^§^41.725795/2.930889	GalA, NS, Mp, Rh and Rg
549	Qar-8a	LBN	^*^34.101944/35.8376	***GalA*** and ***IV***

Country of origin is indicated by ISO 3166 code (https:www.iso.org/iso-3166-coutry-codes.html). Symbols indicate coordinate reliability: ^*^reliable locations; ^§^estimated location (often based on nearby town/city); ^#^no reliable locations within country (corresponds to coordinates for capital city of country of origin). Given outer mucilage traits that were classed as atypical are indicated in normal or bold/italic font if they were significantly higher or lower, respectively, than the global mean absolute values by t-test of p < 0.0001^4^. AV, accession Versailles; GalA, galacturonic acid contents; NS, neutral sugar contents; Mp, molar mass at peak maximum; IV, intrinsic viscosity; Rh, hydrodynamic radius; Rg, radius of gyration.

**Fig. 1:**
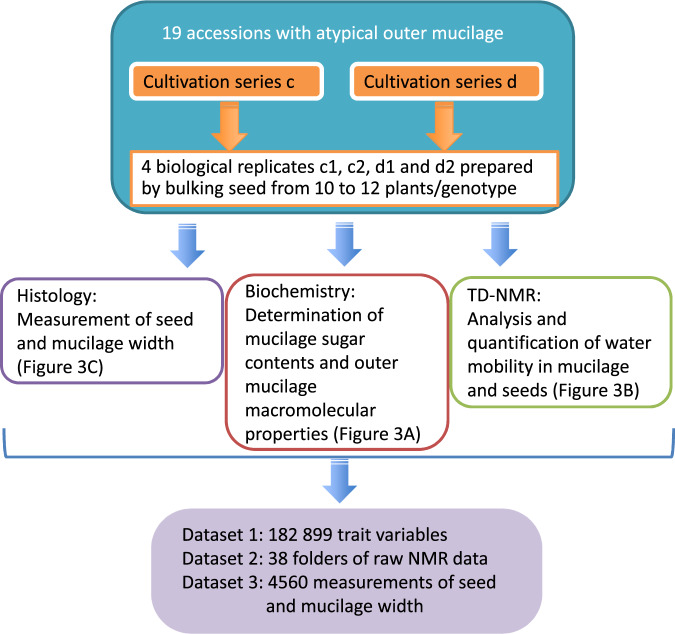
Schematic representation of the production of the datasets 1, 2 and 3 for mucilage and seed traits for Arabidopsis accessions with atypical outer mucilage. Data was generated using four seed lots generated from bulks of independent plants that had been produced at two different times corresponding to series c or d. Analyses of the sugar composition, macromolecular properties, water mobility during imbibition, mucilage and seed width, for the 19 accessions generated raw and treated data available in three datasets.

Histological, biochemical and Time-Domain NMR (TD-NMR) analyses were used to generate three datasets: dataset 1 contains 182 899 variables for 33 mucilage and seed traits, dataset 2 comprises raw NMR data files and dataset 3 is 4560 values measured following microscope acquisition. In addition to confirming previous values for outer mucilage composition and intrinsic viscosity (IV), the size of the hydrated inner mucilage layer, seed and mucilage width were each measured on images acquired following labeling of the seed surface with a cellulose specific stain (DR23) and the periphery of the inner mucilage layer with an antibody recognizing RG-I epitopes (INRA RU1^9^). The amount of the major RG-I sugars in the inner layer was determined following hydrolysis of pectin polymers with rhamnogalacturonan hydrolase. Finally, to obtain information about the mobility of water in interaction with macromolecules in different compartments of seed and mucilage, TD-NMR was carried out on dry seeds and over a period of 23 h of imbibition using either intact seeds or seeds pre-treated to remove outer mucilage. The different steps in data production are summarized in Fig. 1.

## Methods

### Plant material and growth conditions

**Fig. 2:**
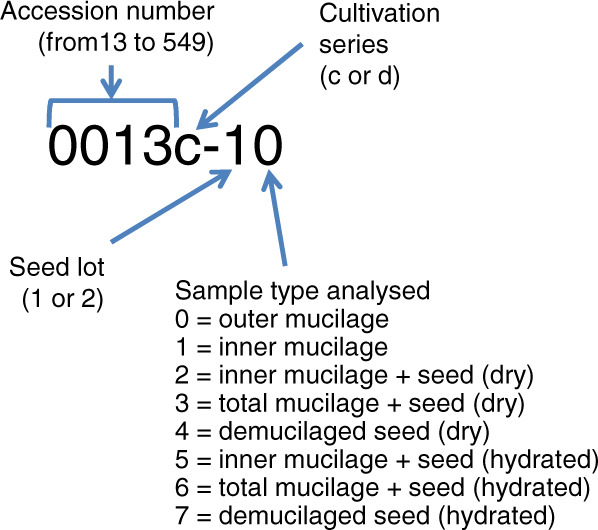
Nomenclature for seed samples analysed to generate dataset.

The 19 accessions used in this study (Table 1) were obtained from the Versailles Arabidopsis stock center (http://publiclines.inra.fr/naturalAccession/index) and are listed by their Versailles identification number in a four-digit format (i.e. 0001 for accession AV1). These were chosen from 306 outlier accessions analysed previously^4^ and included accessions with extreme phenotypes for each of the four macromolecular traits examined, with certain also exhibiting atypical mucilage amounts or composition. Plants were grown in a chamber with 65% relative humidity and 170 µmol m^−2^ s^−1^ of light and for the first three weeks with a 16 h photoperiod at 21 °C and 8 h dark at 18 °C, followed by 6 weeks at 6 °C with an 8 h photoperiod to synchronise flowering when subsequently returned to a 16 h photoperiod. Plants were grown in compost (Tref substrates) following a randomized sowing plan in two independent series of plants grown together, with twenty-four plants of each genotype per series. To differentiate these from the seed stocks produced previously to study outer mucilage traits^3^ these were termed series c, grown from November 2014 to March 2015 and series d from April to August 2015. Four independent biological replicates were produced for analyses by bulking seed harvested from different plants. These were assigned sample codes c1, c2, d1 and d2 corresponding to two independent lots derived from bulks of 10 to 12 plants from series c or d, respectively (Fig. 2). Ten seeds from each lot were weighed using a Sartorius M2P microbalance.

### Biochemistry

**Fig. 3:**
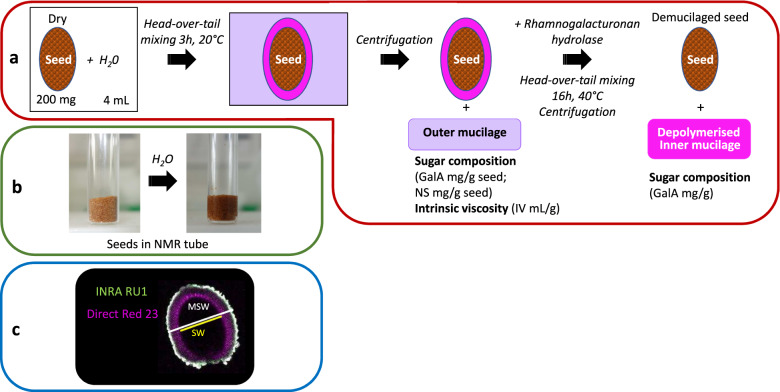
Summary of methodology and experimental workflow used to generate dataset. Extraction procedure used to generate samples for biochemical and NMR analyses (**a**), picture of NMR tubes filled with dried or imbibed seeds (**b**), confocal optical section through a seed immunolabelled with the antibody (INRA RU1) that binds to rhamnogalacturonan 1 with cellulose co-stained with Direct Red 23 showing how mucilage and seed width (MSW) or seed width (SW) were measured (**c**).

Outer mucilage (sample type 0) was water-extracted (4 mL) from seeds (200 mg) and analysed as described previously^3^ (Fig. 3a). Briefly, after 3 h of head-over-tail mixing at 20 °C and centrifugation (8000 *g*, 5 min), water extracts were filtered through a disposable glass microfilter (13 mm diameter, 2.7 μm pore size) and analysed colorimetrically for galacturonic acid (GalA) and total neutral sugar (NS) contents^10,11^. Both quantification methods used are based on the ability of sugars to be converted into furfuric derivates in the presence of hot sulfuric acid. Furfuric derivates can then condense with various phenolic compounds to produce a colored complex that can be quantified colorimetrically. Acidic sugars can be quantified specifically using meta-hydroxy biphenyl (*m*phenyl-phenol or 3 phenyl-phenol)^12^ while neutral sugars can be quantified using orcinol (3,5 dihydroxytoluene)^13^. These methods have been automated^10,11^. Extracts were also analysed for their intrinsic viscosity (IV) by high-performance size exclusion chromatography (HPSEC) coupled to a differential refractometer and a differential pressure viscometer. Seeds remaining after water-extraction were rinsed three times with 8 mL of water and then used to extract inner mucilage (sample type 1) (Fig. 3a). Water volume was adjusted to 2 mL and 2 mL of 100 mM sodium acetate buffer pH 4.5 were added. Twenty µL of rhamnogalacturonan hydrolase at 10 mg mL^−1^ in cold 50 mM sodium acetate buffer pH 4.5 were added. The rhamnogalacturonan hydrolase (EC 3.2.1.171, glycoside hydrolase family 28) used was purified from a technical preparation of *Aspergillus aculeatus* as described in Schols *et al*.^14^. The reaction was incubated at 40 °C for 16 h. After centrifugation (8000 *g*, 5 min), supernatants were carefully removed for GalA analysis by colorimetry.

### Histochemical staining and immunolabeling of inner mucilage

Seed and inner mucilage layer size (Fig. 3c) were determined following immunolabelling and staining of seeds with an anti-RG-I antibody (INRA-RU1^9^) and the cellulose specific fluorescent dye Direct Red 23 (DR23) essentially as previously described^15^, except that 1% (w/v) powdered milk was used for the blocking solution and seeds were mounted for observation directly in the DR23 counterstain. As outer mucilage is lost during the immunolabelling procedure seeds analysed correspond to sample type 5. The INRA-RU1 antibody labels the periphery of the inner mucilage while DR23 labels the cellulose within the inner mucilage and the cell walls on the seed surface. Observations was performed with a Zeiss LSM710 confocal microscope using 488 nm or 561 nm lasers to excite Alexa Fluor 488® or DR23, respectively. Fluorescence emission was detected between 500 and 550 nm for Alexa fluor 488® and 565 and 640 nm for DR23. For each seed lot, measurements were obtained from 30 seeds using Zen software (dataset 3^16^) and the mean value calculated (dataset 1^17^).

### Time-domain NMR

Seeds were either analysed directly (sample type 3 or 6) or after removal of water-soluble mucilage (sample type 2 or 5). The latter were prepared by mixing 350 mg of seeds in 10 mL of water for 3 h at 20 °C. Extracts were then centrifuged at 8000 *g* for 3 min and supernatants carefully removed. Seeds were rinsed four times with 10 mL of water and freeze-dried. Dehydrated seeds with (sample type 3) or without soluble mucilage (sample type 2) were stored at room temperature before being analysed by TD-NMR in dry state or imbibed in water (Fig. 3b).

A Time-Domain spectrometer (Minispec BRUKER, Germany) operating at 0.47 T (resonance frequency of 20 MHz) was used to measure T_2_ relaxation times. The temperature of samples was regulated at 20 °C with a temperature control device (±0.1 °C) connected to a calibrated optical fiber (Optoprim; France). The NMR tubes were filled with dry seeds or dry seeds and water (Fig. 3b) as previously described^18^. Tubes were then weighed and hermetically sealed. Acquisitions of T_2_ were carried out first on dry seeds and then from 3 min (t0) to 23 h (H23) of imbibition. The FID-CPMG sequence used the following parameters: a 90° pulse close to 2.8 µs, a dwell time of 0.4 µs for a FID duration of 150 µs, 16 scans, a recycle delay of 5 s, an echo time of 0.2 ms with 5000 or 16000 data points, depending on the genotype and/or the seed state (dry, with or without soluble mucilage).

Transverse relaxation data were analyzed using the following model:

1 $${{\rm{I}}}_{({\rm{t}})}={\sum }_{i=2}\,{{\rm{I}}}_{{\rm{i}}}\exp {\left(-,\frac{{\rm{t}}}{{{\rm{T}}}_{2{\rm{i}}}}\right)}^{2}+{\sum }_{j=2,4}\,{{\rm{I}}}_{{\rm{j}}}\exp \left(-,\frac{{\rm{t}}}{{{\rm{T}}}_{2{\rm{j}}}}\right)$$

where T_2i_ and T_2j_ are the proton relaxation times of the solid phase of seeds and those of the more mobile populations (water and oil protons), respectively. The corresponding NMR signal intensities were I_i_ and I_j_. Dry seeds samples were characterized by four T_2_ components (T2_1, _2, _3, _4). Compared to previous studies performed on the reference accession Col-0 and two Arabidopsis insertion mutants^18,19^, the present analyses acquired with a longer FID signal, made it possible to identify an additional T_2_ component at around 100 µs for imbibed seeds termed T2_2a while the previously identified component was termed T2_2b so that in total six T2 (T2_1, _2a, _2b, _3, _4, _5) were identified for imbibed seeds. Seeds where outer mucilage had been previously removed by extraction (see above) resulted in the loss of the longer T_2_ relaxation time T2_5 and the splitting of T2_3 into T2_3a and T2_3b (Table 2). Each of these T_2_ components could be assigned to populations of protons in water or oil having different mobilities and proportions^18^.

**Table 2:** Nomenclature used for data in records and indication of sample type analyzed for each trait variable.

Variable	Unit	Variable code	Sample type analyzed	Time points
Galacturonic acid content	mg/g seed	GalA	0 and 1	ND
Neutral sugar content	mg/g seed	NS	0	ND
Intrinsic viscosity	mL/g	IV	0	ND
Mucilage and seed width	µm	MSW	5	ND
Seed width	µm	SW	5	ND
Mass per seed	µg	seed_mass	3	ND
NMR sample mass	g	mass_NMR	2, 3, 5 and 6	ND
Intensity of first T2 component	Arbitrary	I_1	2 and 3	ND
Intensity of first T2 component	Arbitrary	I_1	5 and 6	Yes
Intensity of second T2 component	Arbitrary	I_2	2 and 3	ND
Intensity of T2 component 2a	Arbitrary	I_2a	5 and 6	Yes
Intensity of T2 component 2b	Arbitrary	I_2b	5 and 6	Yes
Intensity of third T2 component	Arbitrary	I_3	2 and 3	ND
Intensity of third T2 component	Arbitrary	I_3	6	Yes
Intensity of T2 component 3a	Arbitrary	I_3a	5	Yes
Intensity of T2 component 3b	Arbitrary	I_3b	5	Yes
Intensity of fourth T2 component	Arbitrary	I_4	2 and 3	ND
Intensity of fourth T2 component	Arbitrary	I_4	5 and 6	Yes
Intensity of fifth T2 component	Arbitrary	I_5	2 and 3	ND
Intensity of fifth T2 component	Arbitrary	I_5	5 and 6	Yes
Time value of T2 component 1	ms	T2_1	2 and 3	ND
Time value of T2 component 1	ms	T2_1	5 and 6	Yes
Time value of T2 component 2	ms	T2_2	2 and 3	ND
Time value of T2 component 2a	ms	T2_2a	5 and 6	Yes
Time value of T2 component 2b	ms	T2_2b	5 and 6	Yes
Time value of T2 component 3	ms	T2_3	2 and 3	ND
Time value of T2 component 3	ms	T2_3	6	Yes
Time value of T2 component 3a	ms	T2_3a	5	Yes
Time value of T2 component 3b	ms	T2_3b	5	Yes
Time value of T2 component 4	ms	T2_4	2 and 3	ND
Time value of T2 component 4	ms	T2_4	5 and 6	Yes
Time value of T2 component 5	ms	T2_5	2 and 3	ND
Time value of T2 component 5	ms	T2_5	5 and 6	Yes

Each variable measured is listed with its corresponding code, unit of measure and the sample type analyzed (for sample code see Fig. 2) including whether acquisition was over multiple time points. ND, not determined.

## Data Records

All three data records use the same sample nomenclature for input and this is explained in Fig. 2 and Table 2.

### Data record 1

The dataset described here contains values obtained from biochemical, microscopy and TD-NMR analyses and has been published on the Data INRAE site^17^. The raw data used to generate mean values for MSW and SW or values for time T2 and intensity I of components 1, 2a, 2b, 3, 4 and 5 at t0 and H23 and for the sample type 6, are available in datasets described in data records 3 and 2, respectively. An overview of the data set is in shown in Table 3 with the following nine columns:

1. sample_code: the sample code (see Fig. 2)
2. accession: the accession number
3. cultivation_series: c or d
4. seed_lot: Biological replicate 1 or 2
5. sample type: the sample type analyzed (0, 1, 2, 3, 5, 6) (see Fig. 2)
6. variable: the code of the variable (see Table 2 for the description)
7. value: the measured value
8. date: the date of acquisition in format (year-month-day) or NA if no time points.
9. time: the time of acquisition in format (hour:minute:second) or NA if no time points

**Table 3:** Overview of the dataset for 33 mucilage and seed traits.

sample_code	accession	cultivation_series	seed_lot	sample_type	variable	value	date	time
0013c-11	13	c	1	1	GalA	5.41	NA	NA
0013c-21	13	c	2	1	GalA	5.39	NA	NA
0013d-11	13	d	1	1	GalA	5.12	NA	NA
0013d-21	13	d	2	1	GalA	5.51	NA	NA
0013c-10	13	c	1	0	GalA	8.86	NA	NA
0013c-20	13	c	2	0	GalA	8.98	NA	NA
0013d-10	13	d	1	0	GalA	8.19	NA	NA
0013c-16	13	c	1	6	T2_1	0.0169	19/01/2016	09:29:52
0013c-16	13	c	1	6	T2_1	0.0178	19/01/2016	09:33:10
0013c-16	13	c	1	6	T2_1	0.0168	19/01/2016	09:36:28
0013c-16	13	c	1	6	T2_1	0.0176	19/01/2016	09:39:47

### Data record 2

Dataset 2 consists of NMR raw data files whose name is the combination of the dataset number, the seed lot (2), the cultivation series (c or d) followed by “imb-“, the imbibition time (t0 or H23) and the accession code. Data correspond to the TD-NMR raw data of intact seeds imbibed in water (code 6) at initial (imb-t0) or final (imb-24H) imbibition time. The format is supplier imposed (.dps) but can be read by any application using tabular formats. The files comprise three columns: the first indicates the total number of recorded data points, the second acquisition time (in ms), while the third column gives the NMR signal intensity (in arbitrary units). Data were recorded using a Time-Domain spectrometer (Minispec BRUKER, Germany) operating at 0.47 T (resonance frequency of 20 MHz) at 20 °C. The Free Induction Decay-Car Purcell Meiboom Gill (FID-CPMG) pulse train acquisition sequence used the following parameters: a 90° pulse close to 2.8 µs, a dwell time of 0.4 µs for a FID duration of 150 µs, 16 scans, a recycle delay of 5 s, an echo time of 0.2 ms with 8000 (imb-t0) or 5000 (imb-24H) data points. The dataset is accessible on the Data INRAE site^19^.

### Data record 3

The values for individual measurements of the two traits seed width and mucilage and seed width used to generate the mean values in dataset 1 are listed as shown in the overview Table 4. In addition to the nine columns found in dataset 1 an additional column indicates the number of the seed measured. The dataset comprises 4560 values corresponding to measurements of 30 seeds from each seed lot. The dataset is accessible on the Data INRAE site^16^.

**Table 4:** Overview of the dataset with individual values for mucilage and seed width (MSW) and seed width (SW).

sample_code	accession	cultivation_series	seed_lot	sample_type	Seed_measured	variable	value	date	time
0013c-15	13	c	1	5	1	MSW	461.51	NA	NA
0013c-15	13	c	1	5	2	MSW	491.71	NA	NA
0013c-15	13	c	1	5	3	MSW	495.34	NA	NA
0013c-15	13	c	1	5	4	MSW	505.35	NA	NA
0013c-15	13	c	1	5	5	MSW	504.27	NA	NA
0013c-15	13	c	1	5	6	MSW	490.55	NA	NA
0013c-15	13	c	1	5	7	MSW	559.38	NA	NA
0013c-15	13	c	1	5	8	MSW	507.29	NA	NA

## Technical Validation

**Fig. 4:**
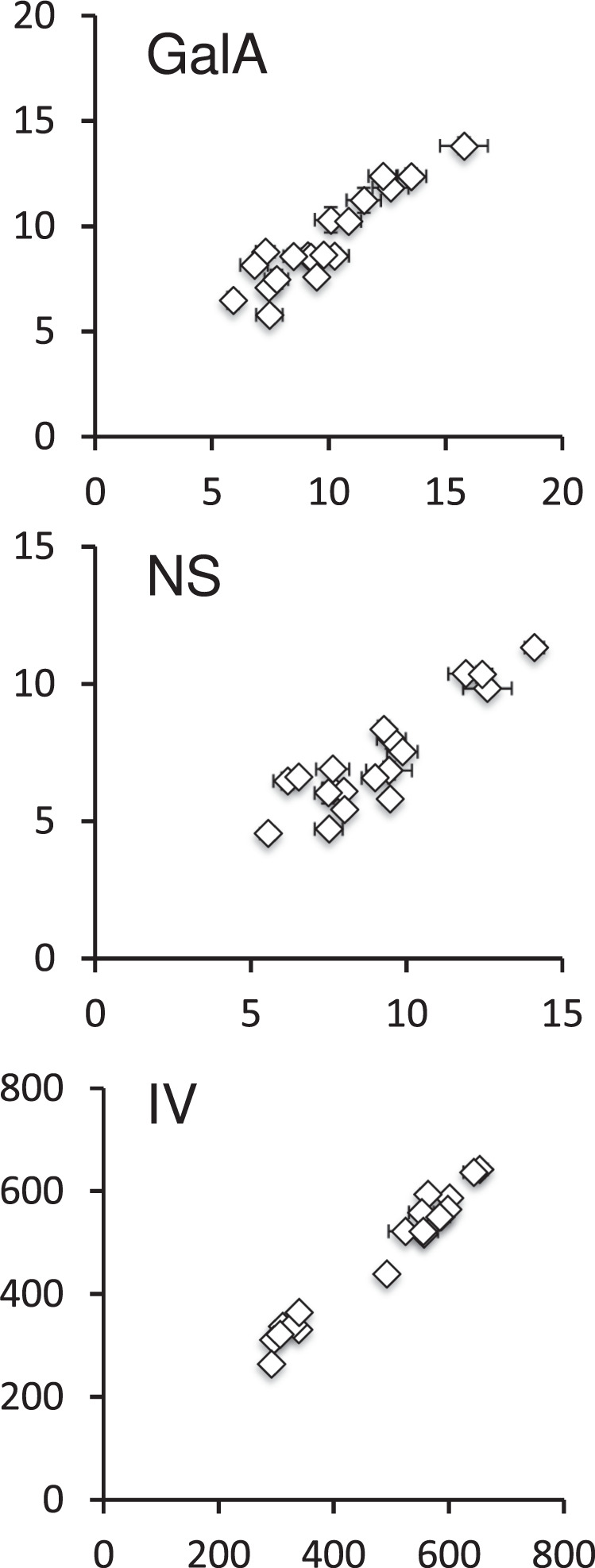
Comparison of outer mucilage variables obtained in this study to previously obtained values to validate the reliability of measurements obtained. Mean values for GalA (mg/g seed), NS (mg/g seed) and IV (mL/g) obtained for samples analysed here (y axis) are plotted versus those previously obtained from seed lots generated from plants cultivated independently for the same accessions^3^ (x axis). Error bars, ±SE (n = 4).

The technical quality of the dataset was validated through the use of four biological replicates of seed lots in the different analyses; replicates were produced from plants cultivated in a randomised format to ensure that any environmental effects from their position within the growth chamber, or from plant neighbours, were minimised. The reproducibility of results was examined for biochemical and histological analyses based on the variation between the four replicates with the highest variation observed being under 7% (Table 5). Furthermore, certain analyses carried out on outer mucilage are equivalent to those previously carried out by Poulain *et al*.^3^, notably for GalA, NS and IV, and these were compared to validate the reliability of measurements and seed lots (Fig. 4). An excellent proportionality between values was observed for all 3 variables with a R^2^ of 0.82, 0.80 and 0.96, respectively. For quantification of sugar concentrations, a standard curve was established using standard solutions of Rha or GalA at 20, 40, 60, 80, and 100 µg/ml, which were measured both before and after a series of samples to confirm technical rigour. HP-SEC columns were calibrated for IV using both a calibrant and a standard sample passed at the beginning, middle and end of a series of samples to check that no drift occurred over time.

**Table 5:** Variation between dataset values from four biological replicates for biochemical and histological variables for the 19 accessions studied is presented as standard errors expressed as a % of the average value of the 4 replicates.

Versailles identification number (AV)	Outer mucilage			Inner mucilage		Seed
	GalA	NS	IV	GalA	Width	Width
0013	2.42	4.86	1.92	1.56	1.18	0.62
0077	3.97	4.37	1.11	1.65	1.16	0.61
0136	2.42	1.08	1.14	2.79	1.14	0.65
0166	3.02	1.80	1.55	1.82	1.12	0.90
0167	5.86	4.41	0.69	3.16	1.14	0.84
0178	2.32	2.09	1.64	3.64	0.85	0.75
0186	1.75	0.90	1.06	3.29	1.15	0.59
0254	4.40	4.81	1.69	4.17	1.01	0.59
0257	1.39	1.92	1.06	1.04	0.88	0.81
0258	4.00	2.90	1.01	4.33	0.97	0.77
0259	5.94	6.25	1.68	4.37	0.77	0.46
0261	2.03	2.48	0.84	2.79	2.52	0.56
0301	1.89	1.75	1.48	4.47	1.10	0.65
0335	1.18	1.06	1.63	3.86	1.08	0.53
0397	6.28	2.94	1.78	2.38	0.87	0.61
0456	5.24	4.00	1.19	2.63	1.33	0.61
0472	3.00	2.85	1.46	2.04	1.11	0.76
0517	2.84	1.98	1.48	6.52	1.62	0.72
0549	3.55	3.53	2.22	2.54	1.39	0.69

GalA, galacturonic acid contents; NS, neutral sugar contents; IV, intrinsic viscosity.

The NMR spectrometer underwent a daily control procedure in accordance with the manufacturer’s recommendations. In addition, the T2 relaxation time and intensity of a reference sample (mineral oil) were controlled each day at spectrometer temperature (around 40 °C). The optical fiber used to regulate sample temperature to 20 °C was calibrated before a series of measurements. In order to validate NMR results, data processing were performed using two different methods that were expected to converge: discrete^20^ and continuous maximum entropy (MEM^21,22^). Each of these methods was performed using two different codes listed below (Table 6). Moreover, the T2 times and amplitudes obtained with Col-0 samples used here were compared for reproducibility with those obtained previously with different Col-0 samples^18^. The reliable acquisition of images by the confocal microscope is certified through annual recalibration of the system, parfocality and light, head scan lens focalisation and collimator by Zeiss, France.

**Table 6:** Versions of software used to acquire and process data.

	Biochemistry	Confocal Microscopy	TD-NMR
Signal acquisition	• San Plus Analyzer from Skalar analytical	• zen version 14,0,15,201	• Bruker the minispec version 2.58Rev.03
	• Viscotek TDA Model 302		
Signal processing	• Flowaccess v3 • Omnisec v1.0	• zen version 8,1,0,484	• Table Curve 2D V5.01 • Scilab-6.0.2

## Data Availability

The different available software and the versions used to acquire and process data presented in the datasets are summarized in Table 6.
